# Comparative efficacy of pediatric atopic dermatitis treatments: a network meta-analysis highlighting dupilumab and pimecrolimus for SCORAD and EASI improvement

**DOI:** 10.3389/fimmu.2026.1676852

**Published:** 2026-05-01

**Authors:** Liu Yang, Ruixue Hu, Yueyuan Wang, Yuanyuan He

**Affiliations:** Department of Pharmacy, Personalized Drug Therapy Key Laboratory of Sichuan Province, Sichuan Academy of Medical Sciences & Sichuan Provincial People’s Hospital, School of Medicine, University of Electronic Science and Technology of China, Chengdu, China

**Keywords:** atopic dermatitis, children, efficacy, network meta-analysis, treatments

## Abstract

**Background:**

Atopic Dermatitis (AD) in children is a common chronic skin condition characterized by dry, itchy and inflamed skin. Various treatments have been applied in the management of AD in children, but the differences in efficacy of different treatment options have not been systematically summarized. The aim of this study was to compare the efficacy of different treatments for atopic dermatitis in children by network meta-analysis.

**Methods:**

We conducted a systematic literature search to select randomized controlled trials (RCTs) that met the inclusion criteria. Databases included PubMed, EMBASE, Cochrane Library, and Web of Science, with a March 10, 2025, search deadline. Certainty of evidence was graded using the CINeMA tool, and risk of bias was assessed using risk of bias 2.0. The efficacy of different treatment regimens was compared using Bayesian network meta-analysis with R software. The primary outcome indicators were the SCORAD (Clinical Score for Atopic Dermatitis) and EASI (Atopic Dermatitis Area and Severity Index) scores.

**Results:**

Thirty-two randomized controlled trials (n=3961) were included. Meta-analysis showed Dupilumab was more effective than Melatonin, Probiotics, SCG, Synbiotic, and Vitamin D for SCORAD. Cumulative probability rankings indicated Dupilumab (96.0%), Tralokinumab (86.8%), and PEC (69.2%) as the top treatments. For EASI, Pimecrolimus, Dupilumab, and Nemolizumab were superior to Probiotics, with Pimecrolimus showing the highest efficacy (99.9%).

**Conclusion:**

Dupilumab may offer greater benefits in reducing SCORAD scores, while Pimecrolimus appears to be more effective for improving EASI. These treatments show potential as favorable options for managing pediatric atopic dermatitis.

**Systematic Review Registration:**

https://www.crd.york.ac.uk/PROSPERO/view/CRD420250650919, identifier CRD420250650919.

## Background

Atopic Dermatitis (AD) is a chronic, relapsing skin disease that usually begins in infancy and early childhood and may vary in clinical presentation with age (1, 2). AD has a profound impact on the physical health and psychological development of children, and in severe cases, not only affects daily life and quality of sleep, but may also cause mental health problems such as anxiety and depression (3, 4). According to the World Health Organization (WHO), AD affects approximately 20% of children globally, and this percentage is particularly high in developed countries (5). With changes in the global environment, lifestyle and immune system mechanisms, the incidence of AD has continued to rise in recent years and has become one of the most common chronic skin diseases in the pediatric population (6). Although the exact etiology of AD is not fully understood, multifactorial interactions are believed to be the main causative mechanism. These factors include genetic susceptibility, immune dysregulation, impaired skin barrier function, and environmental factors (7). Abnormal responses of the immune system, particularly abnormal activation of T-cells, contribute to impaired skin barrier function, which in turn leads to an overreaction of the skin to external stimuli. Together, these mechanisms lead to the typical symptoms of inflammation, dryness, and itching in the skin of AD patients (8).

The main clinical features of AD include dry, erythematous, scaly skin and recurrent itching. These symptoms not only affect the patient’s physical health but also have a significant impact on the patient’s quality of life. Itching is one of the most typical symptoms of AD, which often leads to frequent scratching of the affected area, further aggravating the skin damage and possibly leading to secondary infections (9). During acute episodes of AD, the skin may ooze, break out, and crust, which in severe cases may affect the patient’s appearance, self-esteem, and social interactions, and the chronic relapsing nature of AD means that the patient often experiences recurrent fluctuations in symptoms, leading to a build-up of physical and mental health problems (10). These complications not only exacerbate the patient’s condition but also complicate treatment. AD has long been recognized not only as a localized skin disease, but also as a systemic immune disorder (11). Some patients with AD experience a worsening of the disease, especially when the immune system is affected by other external triggers, which can exacerbate the condition, leading to more severe skin inflammation and the need for long-term medical intervention (12).

Currently, the treatment of AD relies heavily on medication, with common treatments including topical and systemic medications. Topical medications are often the basis of AD management and are effective in relieving symptoms and reducing skin inflammation (13). However, prolonged use of topical medications may result in side effects, especially in pediatric patients, and may affect skin health or produce other adverse reactions. Systemic medications, on the other hand, are often used in patients with moderate-to-severe AD or those for whom topical treatments are ineffective, providing more comprehensive control of the disease and symptomatic relief (14). However, these medications are often accompanied by immunosuppression or other potential side effects, and long-term use may be burdensome to the body. Although medications can be effective in relieving AD symptoms, existing treatments still have limitations. Drug side effects, long-term dependence, and differences in efficacy for different individuals complicate the choice of treatment options. Especially with the chronicity of the disease, how to find appropriate and long-term sustainable treatment options remains a major challenge in clinical treatment. Therefore, rational choice of drug regimens and weighing the relationship between efficacy and side effects are essential for the long-term management of AD patients (15).

With the increase in medication options, especially the introduction of novel drugs such as biologics, clinical treatment options for AD have become diverse. However, most of the existing studies have focused on single comparisons of some specific therapeutic regimens and lacked comprehensive comparisons of multiple therapeutic regimens (16). Therefore, traditional single-comparison studies are unable to fully reflect the differences in efficacy between different drug regimens. In the treatment of AD, network meta-analysis can help compare the efficacy of different drug regimens and provide clinical options for optimal treatment (17). In addition, network meta-analysis can also provide a comprehensive analysis of different efficacy indicators, such as clinical symptom improvement, itch relief and quality of life improvement, which can provide a more comprehensive basis for individualized treatment of patients (18). The main objective of this study is to compare the efficacy of different drug treatments for atopic dermatitis in children through systematic evaluation and network meta-analysis, and to provide a scientific basis for clinical practice. Through this comparison, we hope to identify the most effective treatments and help clinicians make more precise decisions when treating AD in children.

## Methods

This systematic evaluation and meta-analysis will strictly follow the PRISMA (Preferred Reporting Items for Systematic Reviews and Meta-Analyses) guidelines (19). And it is registered in Prospero with registration number CRD420250650919.

### Inclusion and exclusion criteria

Studies included in this study should be randomized controlled trials (RCTs) or open-label trials in children aged 0–18 years with a clear diagnosis of atopic dermatitis (AD), regardless of the severity of the condition, and that provide adequate data on the efficacy of the treatment. The study must include a pharmacologic regimen, including topical and systemic medications such as antihistamines, topical steroids, biologics, etc., and report at least one of the primary clinical outcomes such as SCORAD (Severity of Atopic Dermatitis Score) or EASI (Atopic Dermatitis Area and Severity Index).

Excluded studies include studies from non-clinical trials (observational studies, retrospective studies, case reports) or non-randomized controlled trials of adults (18 years of age or older) or patients without a clear diagnosis of AD, and studies involving only non-pharmacological treatments. In addition, studies that did not report valid outcome metrics or did not provide sufficient data would be excluded. We also excluded studies that were not publicly published or had significant publication bias.

### Literature retrieval

A systematic literature search was conducted to screen randomized controlled trials (RCTs) that met the inclusion criteria. Databases included PubMed, EMBASE, Cochrane Library, and Web of Science, with a March 10, 2025, search deadline. The search terms included Child, Atopic Dermatitis. The specific search strategy is described in **Supplementary Table 1**.

### Data extractions

Two authors independently screened the literature for inclusion by importing the literature into endnote according to the literature inclusion and exclusion criteria, the final included studies were used for data extraction using excel software and if there was a dispute about the literature screening then it would be discussed, or a third person would be sought to adjudicate. The extracted data contained basic characteristics of the study (first author, year of publication, country), basic characteristics of the population (sample size, gender, age), intervention, and outcome.

### Risk of bias

In the Meta-analysis of this study, we used the ROB 2.0 (Risk of Bias 2.0) tool (20) to assess the risk of bias of the included studies. Developed by the Cochrane Collaboration, the ROB 2.0 tool is a standardized tool for assessing the risk of bias in RCTs, which is aimed at systematically identifying and evaluating bias factors that may affect the validity of the results of the study. This improves the accuracy and reliability of Meta-analyses. The ROB 2.0 tool contains five key assessment domains: randomization process, intervention implementation, outcome measures, data reporting, and other sources of bias. Each domain is scored according to the transparency, reasonableness, and potential for bias in the study design and implementation, and is categorized as low risk, high risk, and uncertain risk. In conducting the assessment, two independent reviewers will score each domain based on the specifics of the study. Specifically, the randomization process assesses whether the study adopts an effective random assignment method to avoid selection bias; intervention implementation assesses whether there are deviations from the intended treatment plan that may affect treatment outcomes; outcome measures assess whether standardized, objective, and consistent measurement tools are used to avoid information bias; data reporting assesses whether there is selective reporting bias, which is the practice of reporting only favorable outcomes and ignoring outcomes that do not meet the expected outcomes; and other sources of bias focus on the impact of factors external to the study, such as funding sources and investigator conflicts of interest, on the study. Each area will be assessed based on the specific information in the study report, and if some information is missing or unclear, the reviewers will mark it as “uncertain” and try to resolve it by obtaining further information or communicating with the original authors. Ultimately, the risk of bias for a study will be assessed as low, high, or uncertain based on the results of the assessments in all areas.

### Credibility assessment

In this study, we used the CINeMA (Confidence in Network Meta-Analysis) tool for credibility assessment of evidence (21). CINeMA is a method for systematically assessing the credibility of evidence from studies in network meta-analysis, which is designed to help researchers understand the quality of evidence for different treatment effects in network meta-analysis and thus provide a reliable basis for clinical decision-making. The CINeMA tool assesses the credibility of evidence by considering multiple dimensions, with particular attention to risk of bias, indirectness, inconsistency, network-based relevance, and reporting bias (22).

The CINeMA assessment tool focuses on several dimensions to assess the credibility of evidence. First, the risk of bias assessment considers the risk of bias of each study, using tools such as ROB 2.0 to assess bias in the included randomized controlled trials (RCTs). By assessing the reasonableness of the study design, especially the potential bias in the randomization process, intervention implementation, outcome measurement, and data reporting, CINeMA can help us to identify and control the bias factors to improve the credibility of the evidence. Second, indirectness assesses the comparability between different studies, especially the heterogeneity in terms of intervention, participants, and outcome measures. If there are large differences in intervention protocols, study populations, or outcome measures across studies, this may affect the applicability of the evidence and reduce credibility. When conducting indirectness assessment, we pay special attention to the applicability of the studies and extrapolation of the results. Third, inconsistency assesses whether treatment effects in a network are consistent across studies. Network Meta-analyses may have inconsistent treatment effects by pooling multiple direct and indirect comparisons together. If there are significant differences in the results of comparisons between treatment regimens in a network, this indicates inconsistency, which can reduce the credibility of the evidence. In assessing inconsistency, we will examine whether there are heterogeneous or inconsistent effects between treatment regimens and thus assess the robustness of the network results. Fourth, network-based assessment of relevance involves factors such as sample size, event rates, and the width of confidence intervals. Insufficient sample size or low event rates for certain comparisons in the network may lead to imprecision in the results, thereby reducing the credibility of the evidence. The assessment of this dimension helped us to identify treatment effects that may be subject to uncertainty due to insufficient sample size or incomplete data. Finally, reporting bias considers whether there is publication bias in the included studies, specifically meaning that those studies with significant outcomes are more likely to be published, while those without significant outcomes may not have been included. During the CINeMA assessment process, we consider whether there is a risk of studies not being published or selectively reporting outcomes, which may affect the comprehensiveness and objectivity of the evidence.

### Statistical analysis

In this study, statistical analysis will be performed using a Bayesian framework for Network Meta-Analysis (NMA) (23) to compare the efficacy of different treatments in pediatric atopic dermatitis. The primary outcome indicators include SCORAD and EASI, First, a treatment network will be constructed by connecting studies that directly or indirectly compare two or more treatments. Each treatment will serve as a node, and edges between nodes represent direct comparisons between treatments performed in individual studies. Next, Bayesian network Meta-analysis was employed, using mean difference (MD) as an indicator of treatment effect for continuous outcomes, a Bayesian approach allowing the introduction of prior distributions and the use of Markov chain Monte Carlo (MCMC) methods to generate posterior distributions of treatment effects. In this study, Deviance Information Criterion (DIC) will be used to assess the consistency of the network Meta-analysis model. Specifically, two models will be constructed: one assuming consistency and the other assuming inconsistency. By comparing the DIC values of these two models, we can determine whether there is inconsistency in the network. To account for inter-study heterogeneity, a random effects model will be used for the analysis. Heterogeneity will be assessed by the I² statistic, with significant heterogeneity indicated if the I² value exceeds 50%. Network consistency will also be tested to ensure consistency between direct and indirect evidence. If inconsistency is found, possible causes will be explored, and the model will be adjusted through sensitivity analysis. All statistical analyses will be performed using R software (version 4.0.0) and the “gemtc” package, which is specifically designed for Bayesian network meta-analysis. The results will be presented in the form of a posteriori means, confidence intervals (95% CrI) and ranking probabilities, thus providing reliable conclusions on the comparison of treatments.

## Results

### Literature search results

**Figure 1:**
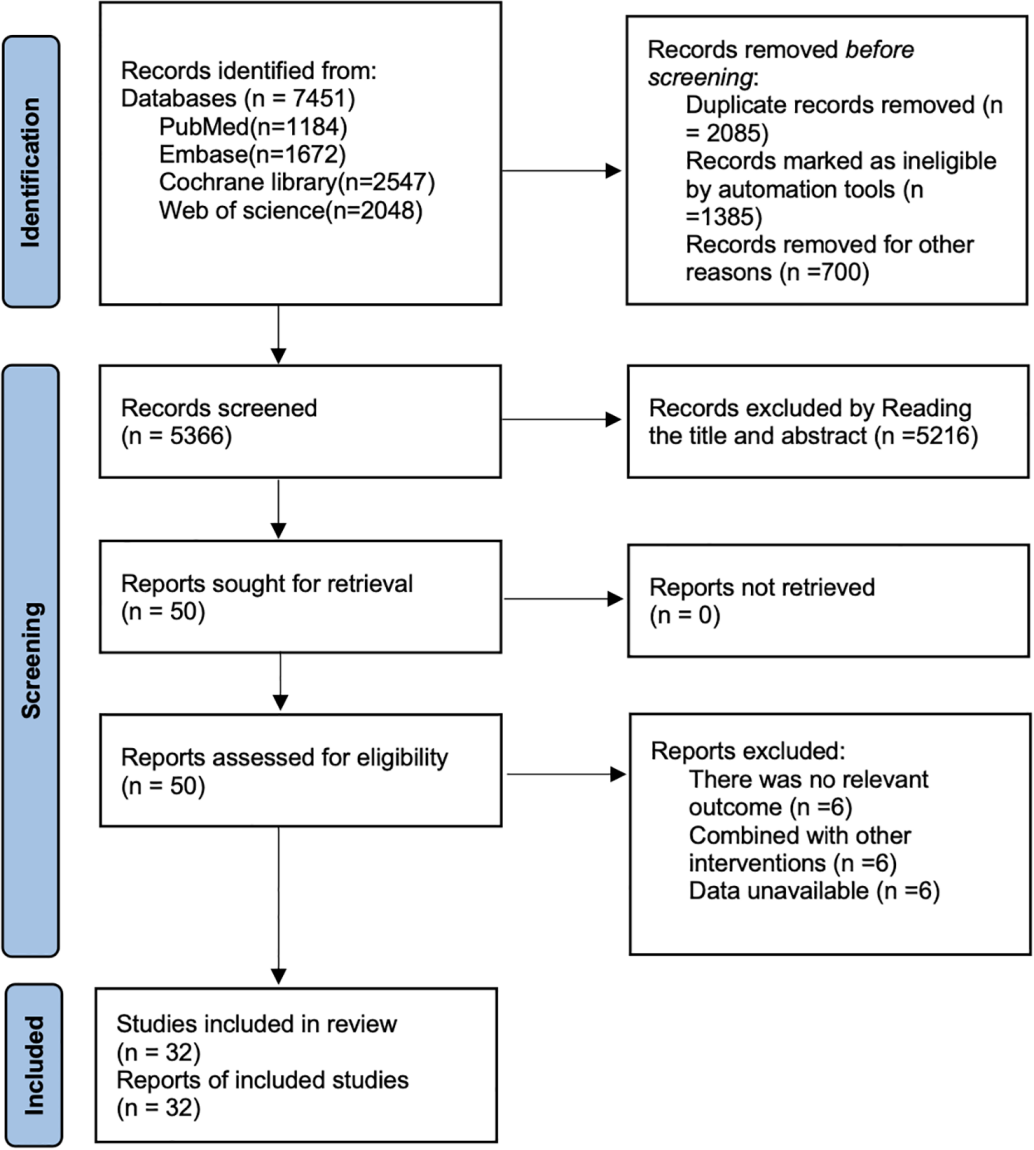
Literature search flow chart.

A total of 7451 articles were retrieved by searching databases [PubMed (n=1184), Embase (n=1672), Cochrane library (n=2547), Web of science (n=2048)], by removing duplicates 2085, by reading titles and abstracts 5216, by reading full text removal of 18, and finally 32 articles (24–55) were included. The literature search flow chart is shown in **Figure 1**.

### Basic characteristics of included studies

Thirty-two randomized controlled studies were included in this study, comprising 3961 patients, with interventions containing plant extract cream (PEC); sodium cromoglicate (SCG); vitamin D (VD); eicosatetraenoic acid (EPA); probiotics; melatonin; omalizumab; pimecrolimus; synbiotic; dupilumab; difamilast; baricitinib; nemolizumab; tralokinumab; abrocitinib, and the specific basic characteristics are shown in **Table 1**.

**Table 1:** Table of basic characteristics.

Study	Year	Country	Sample size	Gender(M/F)	Mean age	Intervention	Outcomes
Abbasi	2017	Iran	PEC:16 Control:15	17/14	PEC:2.35 Control:2.13	PEC: Fig fruit extract 8%	SCORAD
Ahn	2020	Korea	Probiotics:41 Control:41	37/45	Probiotics:4.8 Control:5.4	Probiotics: L. pentosus 1X10^10^	SCORAD
Ardakani	2018	Iran	Melatonin:35 Control:35	34/36	Melatonin:8.4 Control:8.9	6 mg melatonin	SCORAD
Berth	2015	UK	SCG:103 Control:103	100/106	SCG:5.2 Control:5.5	4% cutaneous emulsion of sodium cromoglicate	SCORAD
Borzutzky	2024	Chile	VD:53 Control:48	51/60	VD:5.8 Control:6.9	VD 400 IU	SCORAD
Chan	2024	UK	Omalizumab:30 Control:32	32/30	Omalizumab:10.2 Control:10.4	Omalizumab 150mg	SCORAD; EASI
Chang	2016	China	Melatonin:24 Control:24	25/23	Melatonin:7.6 Control:7.3	3 mg melatonin	SCORAD
Edwards	2015	UK	SCG:118 Control:59	94/83	SCG:5.4 Control:4.6	4% cutaneous emulsion of sodium cromoglicate	SCORAD
Eichenfield	2002	USA	Pimecrolimus:267 Control:136	202/201	1-17	Pimecrolimus 1%	EASI
Farid	2011	Iran	Synbiotic:19 Control:21	25/15	Synbiotic:1.23 Control:1.12	Lactobacillus casei,	SCORAD
Feito	2023	Spain	Probiotics:35 Control:35	24/46	Probiotics:8.4 Control:8.43	Probiotics: L. pentosus 1X10^10^	SCORAD
Folster	2006	Germany	Probiotics:26 Control:27	34/19	Probiotics:1.4 Control:1.9	Probiotics: Lactobacillus rhamnosus	SCORAD
Gerasimov	2010	Ukraine	Probiotics:43 Control:47	56/34	Probiotics:2.12 Control:2.23	Lactobacillus acidophilus	SCORAD
Gruber	2007	Charit	Probiotics:54 Control:48	69/33	Probiotics:7.7 Control:7	Probiotics: Lactobacillus rhamnosus	SCORAD
Han	2012	Korea	Probiotics:58 Control:60	69/49	Probiotics:4.6 Control:5.1	Probiotics: Lactobacillus rhamnosus	SCORAD
Jeong	2022	Korea	Probiotics:33 Control:33	30/36	Probiotics:5.67 Control:5.33	Lactobacillus rhamnosus	SCORAD
Lara	2019	Canada	VD:21 Control:24	24/21	VD:8.1 Control:8.5	VD 400 IU	SCORAD
Meysami	2021	Iran	PEC:22 Control:22	21/23	PEC:1.43 Control:1.34	PEC: Malva sylvestris L	SCORAD
Mirrahimi	2023	Iran	EPA:23 Control:23	25/21	EPA:3.23 Control:3.56	250 mg eicosapentaenoic acid	SCORAD
Navarro	2018	Spain	Probiotics:26 Control:24	24/26	Probiotics:9.35 Control:8.96	Probiotics: Lactobacillus rhamnosus	SCORAD
Niseteo	2024	Croatia	EPA:26 Control:26	21/31	EPA:1.8 Control:2.3	250 mg eicosapentaenoic acid	SCORAD
Paller	2024	USA	Dupilumab:63 Control:62	79/56	Dupilumab:3.9 Control:3.9	Dupilumab: 200/300mg	SCORAD
SAEKI	2020	Japan	Difamilast:49 Control:24	NR	2-14	Difamilast: 0.3%-1%	EASI
Shafiei	2011	Iran	Synbiotic:18 Control:18	24/12	Synbiotic:14.7 Control:15.4	Synbiotic: probiotic plus prebiotic	SCORAD
Simpson	2020	USA	Dupilumab:166 Control:85	148/103	Dupilumab:14.4 Control:14.5	Dupilumab: 200/300mg	SCORAD; EASI
Torrelo	2023	Spain	Baricitinib:361 Control:120	339/242	2-18	Baricitinib:1-3mg	EASI
Wu	2017	China	Probiotics:33 Control:33	44/22	Probiotics:1.5 Control:1.8	Probiotics: Lactobacillus rhamnosus	SCORAD;
Yang	2014	Korea	Probiotics:50 Control:50	53/47	Probiotics:4.12 Control:3.97	Probiotics: Lactobacillus rhamnosus	EASI
Igarashi	2024	Japan	Nemolizumab:45 Control:44	53/36	Nemolizumab:9.2 Control:8.9	Nemolizumab:30mg	EASI
Paller	2023	USA	Tralokinumab:195 Control:94	149/140	10-17	Tralokinumab:150mg/300mg	SCORAD; EASI
Silverberg	2010	Netherlands	Symbiotic:46 Control:44	59/31	Symbiotic:5 Control:4.8	Synbiotic: probiotic plus prebiotic	SCORAD;
Eichenfield	2021	USA	Abrocitinib:189 Control:96	145/140	15	Abrocitinib:100/200mg	EASI

PEC, Plant extract cream; SCG, Sodium Cromoglicate; VD, vitamin D; EPA, Eicosapentaenoic Acid; SCORAD, scoring atopic dermatitis; EASI, Eczema Area and Severity Index.

### Risk of bias results

ROB2.0 was used for quality assessment in this study, the study of 1 included article (53) did not account for the specific method of randomization and was therefore assessed as unclear, the rest of the studies clearly accounted for the method of randomization and were therefore assessed as low-risk, and the results of the specific quality assessment are shown in (**Supplementary Figures 1**, **2**). For all outcomes, the overall certainty of the evidence was low. For SCORAD, EASI, we assessed the level of confidence in the evidence compared with the control group, with 57.9% of the evidence rated as low or very low (**Supplementary Table 2**).

### Results of consistency modeling

The current study used a random-effects model to compare the difference in DIC between consistent and inconsistent modeling, and the absolute value of the difference in DIC was <5, The results (**Supplementary Table 3**) indicate that SCORAD and EASI’s have consistency.

### SCORAD

**Figure 2:**
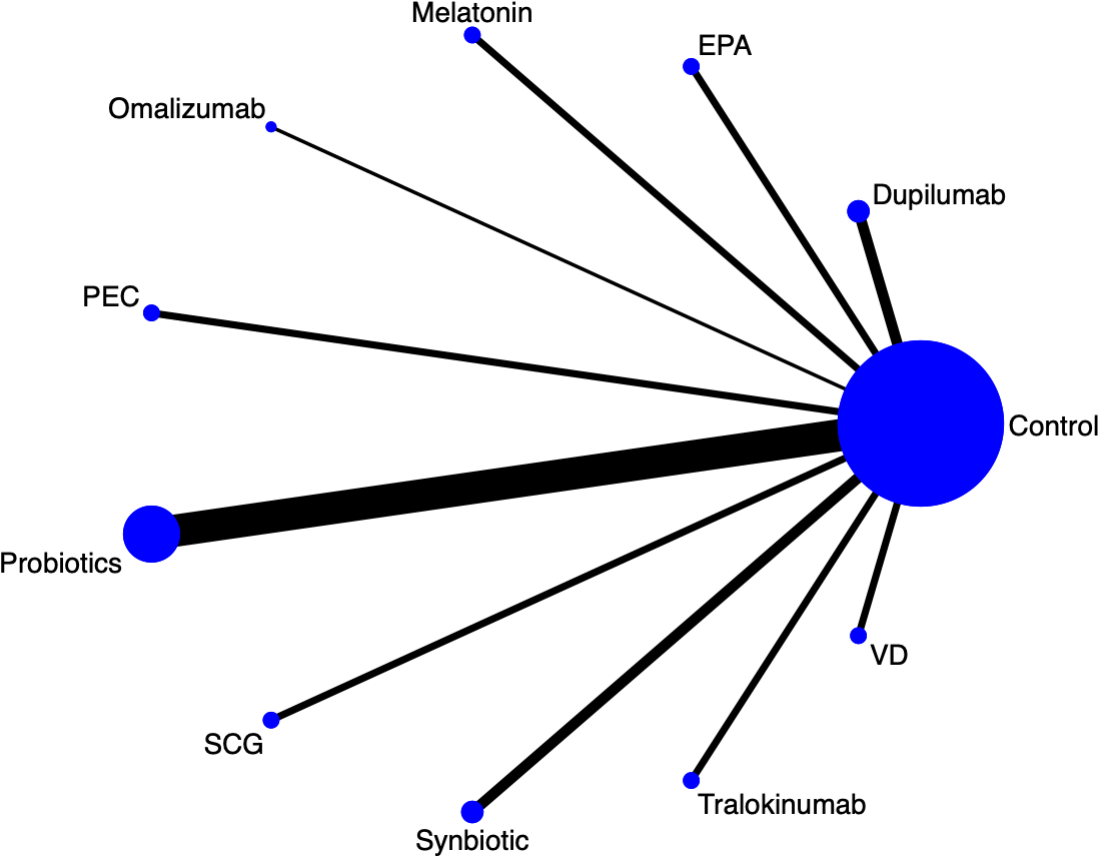
SCORAD network diagram.

**Figure 3:**
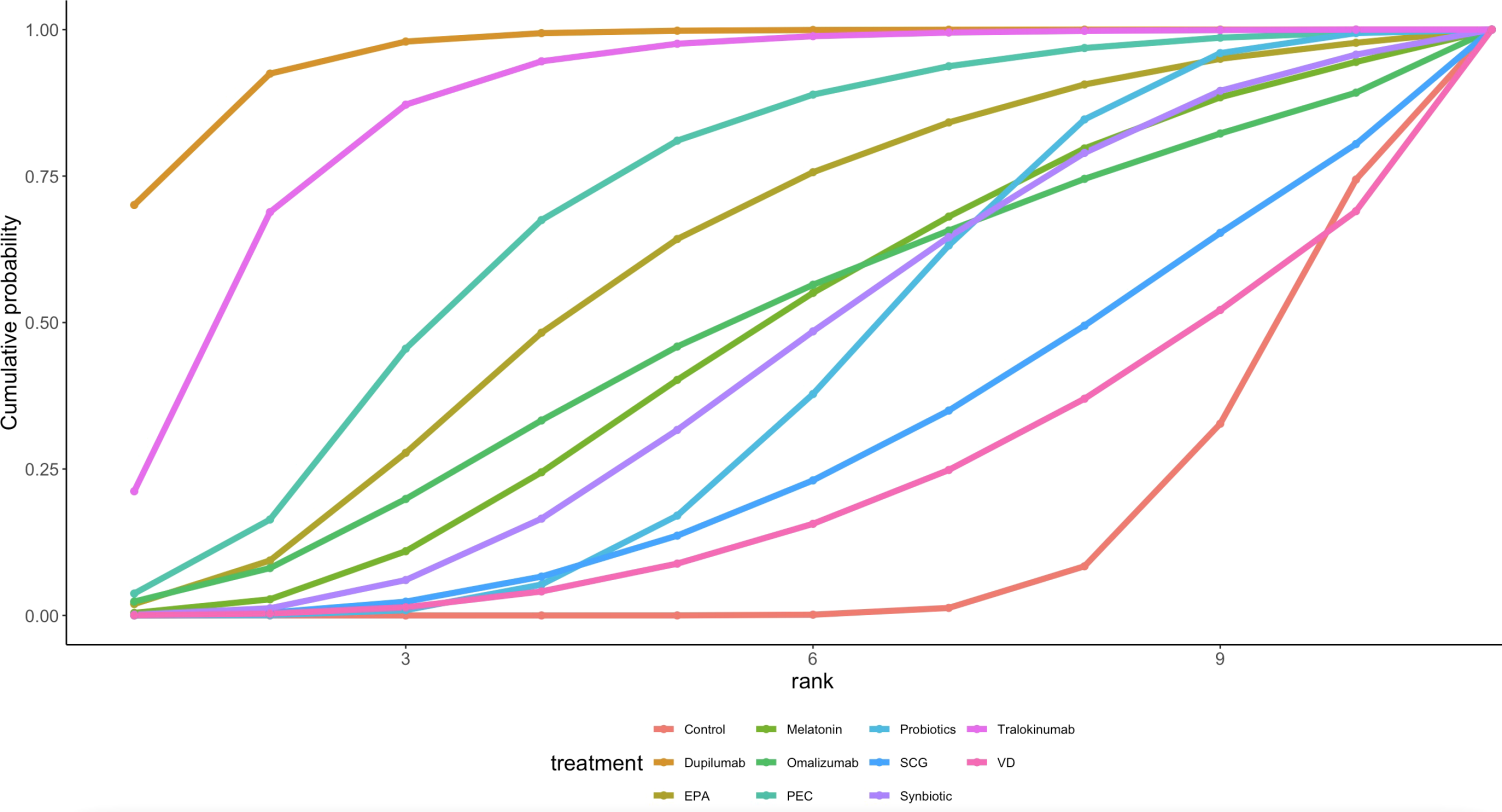
Ranking graph of SCORAD cumulative probability.

Twenty-eight articles in the current study mentioned SCORAD, and the network diagram (**Figure 2**) suggests that PEC; SCG; VD; eicosatetraenoic acid (EPA); probiotics; melatonin; omalizumab; symbiotic; dupilumab; tralokinumab A direct comparison was formed with control. A league table (**Table 2**) suggested that Dupilumab was superior to Melatonin [MD=-16.54, 95%CI (-30.46, -2.42)],Probiotics [MD=-18.03, 95%CI (-27.89, -7.79)],SCG [MD=-20.77, 95% CI (-34.34, -6.98)], Synbiotic [MD=-17.35, 95%CI (-29.61, -4.19)], and VD [MD=-22.32, 95%CI (-36.04, -8.23)]. The cumulative probability ranking by area under the curve (**Table 3**, **Figure 3**) revealed Dupilumab (96.0%), Tralokinumab (86.8%), PEC (69.2%), and Control (11.7%).

**Table 2:** SCORAD League table.

MD 95%CrI										
Control										
23.36 (14.56, 31.89)*	Dupilumab									
10.05 (-1.8, 21.89)	-13.33 (-27.9, 1.54)	EPA								
6.82 (-4.33, 17.82)	-16.54 (-30.46, -2.42) *	-3.2 (-19.49, 13.01)	Melatonin							
7.31 (-8.28, 22.86)	-16.05 (-33.79, 1.98)	-2.75 (-22.34, 16.82)	0.5 (-18.59, 19.74)	Omalizumab						
12.52 (1.82, 23.78) *	-10.83 (-24.43, 3.54)	2.49 (-13.38, 18.84)	5.7 (-9.61, 21.59)	5.21 (-13.48, 24.55)	PEC					
5.34 (0.35, 10.47) *	-18.03 (-27.89, -7.79) *	-4.69 (-17.59, 8.23)	-1.48 (-13.53, 10.81)	-1.97 (-18.28, 14.53)	-7.2 (-19.5, 4.64)	Probiotics				
2.58 (-8.06, 13.22)	-20.77 (-34.34, -6.98) *	-7.47 (-23.41, 8.48)	-4.24 (-19.54, 11.1)	-4.69 (-23.67, 14.11)	-9.95 (-25.59, 5.14)	-2.76 (-14.58, 8.96)	SCG			
6.02 (-2.98, 15.59)	-17.35 (-29.61, -4.19) *	-4.01 (-18.79, 11.23)	-0.8 (-14.91, 13.96)	-1.27 (-19.11, 17.16)	-6.51 (-20.91, 7.78)	0.68 (-9.68, 11.49)	3.44 (-10.41, 17.8)	Synbiotic		
18.79 (8.55, 28.93) *	-4.6 (-17.87, 9.06)	8.75 (-6.98, 24.47)	11.95 (-3.06, 27.13)	11.49 (-7.14, 30.13)	6.27 (-9.06, 20.95)	13.45 (1.95, 24.77) *	16.2 (1.38, 30.92) *	12.76 (-1.31, 26.25)	Tralokinumab	
1.04 (-9.77, 11.93)	-22.32 (-36.04, -8.23) *	-9 (-25.05, 7)	-5.79 (-21.18, 9.79)	-6.23 (-25.2, 12.72)	-11.49 (-27.22, 3.68)	-4.3 (-16.32, 7.65)	-1.56 (-16.66, 13.72)	-5 (-19.52, 9.07)	-17.75 (-32.63, -2.76) *	VD

*Means P<0.05 PEC, Plant extract cream; SCG, Sodium Cromoglicate; VD, vitamin D; EPA, Eicosatetraenoic Acid.

**Table 3:** Cumulative probability ranking table.

Intervention	SCORAD (%)	EASI (%)
Control	11.7	8.60
Dupilumab	96.0	81.9
EPA	59.5	NR
Melatonin	46.5	NR
Omalizumab	47.8	60.6
PEC	69.2	NR
Probiotics	40.4	3.70
SCG	27.6	NR
Synbiotic	43.3	NR
Tralokinumab	86.8	58.8
VD	21.3	NR
Abrocitinib	NR	47.0
Baricitinib	NR	23.2
Difamilast	NR	37.4
Nemolizumab	NR	78.8
Pimecrolimus	NR	99.9

### EASI

**Figure 4:**
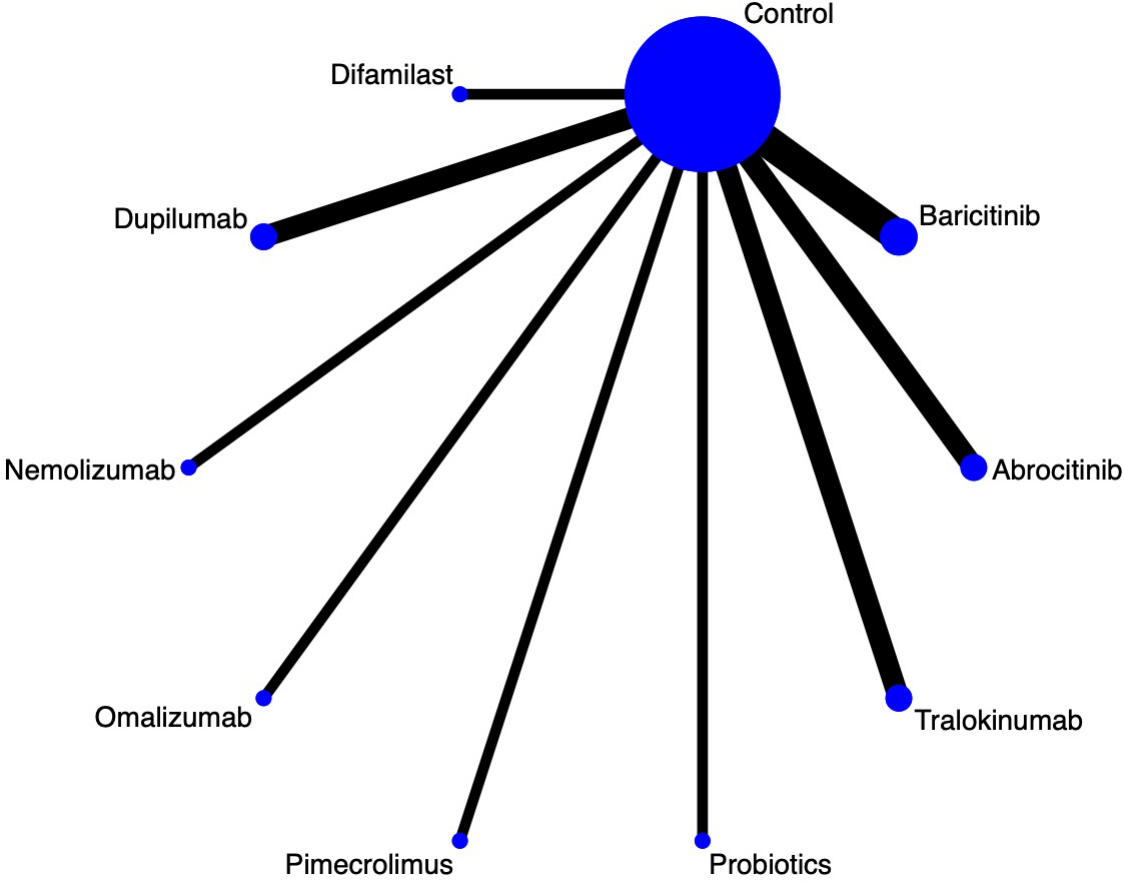
EASI network diagram.

**Figure 5:**
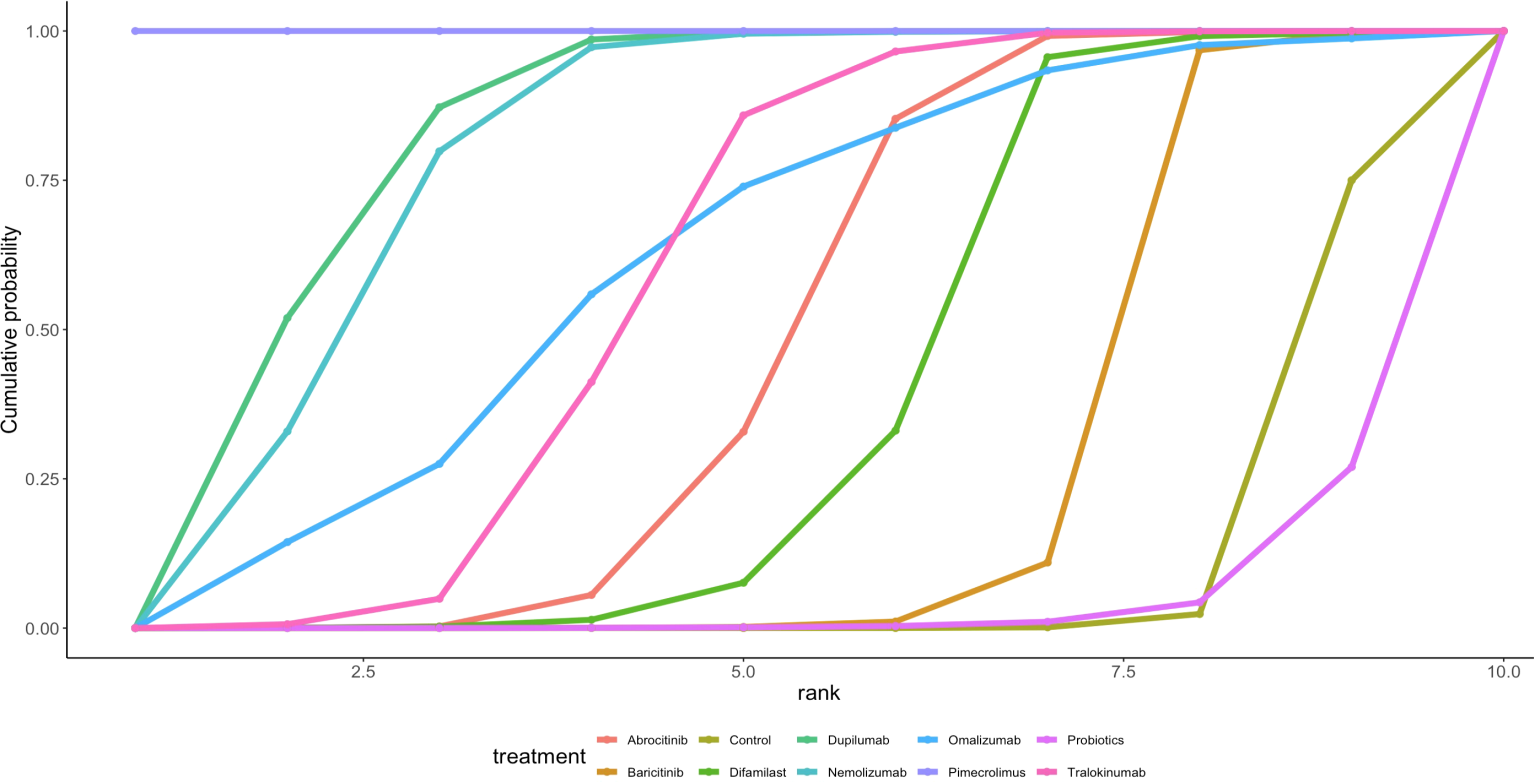
Ranking graph of EASI cumulative probability.

Fourteen articles in this study mentioned EASI, and the network diagram (**Figure 4**) suggests that baricitinib; abrocitinib; tralokinumab; probiotics; pimecrolimus; omalizumab; dupilumab; nemolizumab and difamilast form a direct comparison with control. It was suggested by league table (**Table 4**) that Difamilast was superior to Probiotics [MD=-4.92, 95%CI (-8.52, -1.27)], Dupilumab was superior to Probiotics [MD=-12.26, 95%CI (-16.6, -7.88)], and Nemolizumab was superior to Probiotics [MD=-11.64, 95%CI (-16.01, -7.26)], Omalizumab was superior to Probiotics [MD=-8.6, 95%CI (-16.59, -0.62)], Pimecrolimus was superior to Probiotics [MD=-40.18, 95%CI (-44.92, -35.46)]. Cumulative probability ranking by area under the curve (**Table 3**, **Figure 5**) found Pimecrolimus (99.9%), Dupilumab (81.9%), Nemolizumab (78.8%), and Probiotics (3.7%).

**Table 4:** EASI League table.

MD 95%CrI									
Abrocitinib									
-3.29 (-5.79, -0.78)*	Baricitinib								
-5.32 (-7.43, -3.19) *	-2.03 (-3.4, -0.65) *	Control							
-1.16 (-4.32, 2.01)	2.13 (-0.62, 4.87)	4.16 (1.76, 6.55) *	Difamilast						
6.18 (2.15, 10.18) *	9.48 (5.82, 13.1) *	11.5 (8.07, 14.88) *	7.35 (3.22, 11.43) *	Dupilumab					
5.56 (1.57, 9.59) *	8.84 (5.2, 12.52) *	10.87 (7.47, 14.3) *	6.71 (2.6, 10.88) *	-0.63 (-5.44, 4.24)	Nemolizumab				
2.53 (-5.27, 10.35)	5.82 (-1.83, 13.47)	7.85 (0.33, 15.38) *	3.69 (-4.16, 11.56)	-3.66 (-11.9, 4.69)	-3.03 (-11.33, 5.25)	Omalizumab			
34.1 (29.75, 38.5) *	37.39 (33.32, 41.47) *	39.42 (35.58, 43.28) *	35.26 (30.79, 39.73)	27.93 (22.82, 33.06)	28.55 (23.39, 33.66)	31.56 (23.13, 40.08) *	Pimecrolimus		
-6.08 (-9.54, -2.59) *	-2.79 (-5.83, 0.29)	-0.76 (-3.49, 2)	-4.92 (-8.52, -1.27) *	-12.26 (-16.6, -7.88) *	-11.64 (-16.01, -7.26) *	-8.6 (-16.59, -0.62) *	-40.18 (-44.92, -35.46) *	Probiotics	
1.81 (-1.95, 5.59)	5.1 (1.7, 8.51)	7.12 (4, 10.26)	2.97 (-0.94, 6.87)	-4.37 (-9, 0.28)	-3.75 (-8.37, 0.89)	-0.72 (-8.84, 7.45)	-32.3 (-37.31, -27.32) *	7.88 (3.73, 12.04) *	Tralokinumab

*Means P<0.05 PEC, Plant extract cream; SCG, Sodium Cromoglicate; VD, vitamin D; EPA, Eicosatetraenoic Acid.

### Subgroup analysis

This study conducted subgroup analyses based on treatment regimens. Results (**Supplementary Figures 3**, **S4**) indicate that for SCORAD, systemic therapy [MD = -8.87, 95% CI (-13.72, -4.04)] and topical therapy [MD = -9.19, 95% CI (-16.09, -2.82)] showed significant differences. For EASI, systemic therapy [MD = -3.79, 95% CI (-4.76, -2.82)] and topical therapy [MD = -13.44, 95% CI (-23.64, -3.24)].

### Publication bias

The current study detected their publication bias through funnel plots, and the results (**Supplementary Figures 5**, **6**) funnel graphs were symmetrical to each other, indicating that the presence of publication bias was less likely.

## Discussion

This study is the first to explore the effectiveness of different treatment modalities, for AD in children, using network meta-analysis, which found that for SCORAD, Dupilumab (96.0%) > Tralokinumab (86.8%) > PEC (69.2%). For EASI, Pimecrolimus (99.9%)>Dupilumab (81.9%)>Nemolizumab (78.8%). Dupilumab may be superior in reducing SCORAD scores, while Pimecrolimus appears to be more effective in improving EASI.

The SCORAD score is an important tool for assessing the clinical efficacy of atopic dermatitis, which mainly involves the dimensions of skin damage, itchiness and sleep quality. In this study, Dupilumab was significantly superior to Melatonin, Probiotics, SCG, Synbiotic, and VD, demonstrating a significant advantage in relieving skin symptoms and improving quality of life. This is consistent with previous studies (56, 57), where Dupilumab has been shown to be efficacious in the treatment of moderate-to-severe atopic dermatitis in several clinical trials. For example, Wollenberg et al. (2020) showed that Dupilumab was effective in reducing AD symptoms and provided significant relief of skin damage and itching (58). The performance of Tralokinumab and PEC should not be overlooked, with cumulative probability rankings of 86.8% and 69.2%, respectively. Although the efficacy of these biologics is slightly inferior to Dupilumab, they still provide an important clinical alternative, especially in the treatment of patients who are ineffective on Dupilumab, and the effectiveness of Tralokinumab as an IL-13 antagonist has gained increasing attention. This is consistent with the findings of Beck et al. (2023) on Tralokinumab showing significant efficacy in AD treatment (59), suggesting that IL-13 is a non-negligible target in AD treatment.

The EASI score is another clinical scale commonly used to assess the efficacy of atopic dermatitis treatment, which evaluates the area and severity of skin damage. Pimecrolimus showed the most significant efficacy in the EASI score, with a cumulative probability ranking of 99.9%. The superiority of Pimecrolimus compared to Dupilumab (81.9%) suggests that despite the strong clinical efficacy advantage of Dupilumab, the topical therapeutic agent pimecrolimus has an irreplaceable role to play in patients with mild to moderate AD, pimecrolimus is commonly used as a topical immunosuppressant in the treatment of Pimecrolimus, as a topical immunosuppressant, is commonly used in patients with mild to moderate AD, and has been shown to be excellent in relieving skin inflammation and minimizing drug side effects (60).

Interestingly, this study showed that Probiotics were significantly less effective than other treatments. Despite the potential of probiotics in regulating intestinal microecology and enhancing immune tolerance, their efficacy in atopic dermatitis is still controversial. Similar studies, such as Sun et al.’s (2023) (61) Meta-analysis of probiotics in the treatment of AD, noted that although probiotics may help alleviate symptoms, their efficacy is usually mild and lacks support for long-term effectiveness. Therefore, probiotics have limited efficacy as a single treatment in the clinical management of AD and may need to be used in combination with other treatment modalities.

The efficacy of Dupilumab and Tralokinumab can be attributed to their inhibitory effects on the key cytokines in the immune system, IL-4 and IL-13, which are key cytokines in the Th2-type immune response, and which trigger chronic inflammatory responses and disruption of the barrier function of the skin through activation of the IL-4Rα and IL-13Rα receptors (62, 63). Dupilumab and Tralokinumab effectively attenuated the immune overreaction, reduced skin inflammation, and improved skin barrier function in AD patients by inhibiting the IL-4/IL-13 signaling pathway, respectively. In contrast, Pimecrolimus, as a topical immunosuppressant, attenuates the inflammatory response mainly by inhibiting T-cell activation, and its mechanism of action is different from that of the biologics, but it is equally effective in reducing local inflammation and relieving symptoms (64). Despite its relatively mild action, Pimecrolimus can avoid systemic side effects due to its topical therapeutic character and is particularly suitable for mild to moderate patients (65).

The results of this study have important implications for clinical treatment. First, Dupilumab, as a biologic agent targeting the IL-4/IL-13 signaling pathway, demonstrated superior efficacy, especially in patients with severe AD, and it significantly improved skin symptoms and patients’ quality of life. In clinical practice, Dupilumab should be regarded as the treatment of choice for patients with severe or recalcitrant AD, and its higher efficacy with lower side effects provides patients with better therapeutic expectations. Secondly, Tralokinumab, as an IL-13 antagonist, has shown more significant efficacy, especially in patients who cannot tolerate or respond poorly to Dupilumab, Tralokinumab may become an important alternative treatment option. Considering the high cost of Dupilumab and Tralokinumab, Pimecrolimus as a topical agent remains the first choice for the treatment of mild-to-moderate AD in families with limited financial resources, and its good tolerability and low side effects make it a safe and effective treatment option.

Our subgroup analyses based on treatment regimens revealed notable findings. For SCORAD, both systemic therapy and topical therapy were associated with significant improvements, suggesting that both approaches effectively reduce overall disease severity in pediatric atopic dermatitis. Interestingly, for EASI, while systemic therapy demonstrated a moderate benefit, topical therapy showed a larger effect, highlighting that certain topical treatments, such as pimecrolimus, may be particularly effective in improving lesion-specific outcomes. These results underscore the importance of tailoring treatment strategies according to disease severity and specific clinical endpoints. Moreover, the comparable efficacy of systemic agents like dupilumab and targeted topical therapies suggests flexibility in therapeutic decision-making, allowing clinicians to balance efficacy with safety and patient preference.

### Strengths and limitations

1. This study utilized a network Meta-analysis (NMA) methodology, which can compare multiple treatment options simultaneously, providing a comprehensive and direct comparison of different treatment modalities. This method allows for the combination of direct and indirect evidence, increasing the generalizability and reliability of the results. By comparing the efficacy of various treatments, the study provides clinicians with a broad perspective on different treatment options and can inform decision-making, especially when faced with patients with different treatment responses.
2. Two widely used clinical assessment tools, the SCORAD and the EASI, which have been extensively validated in clinical practice and research, were used in the study to ensure the clinical relevance of the findings. the SCORAD and EASI scales, which respectively take multiple dimensions (skin lesions, itchiness, sleep quality) Assessing the severity of the disease makes the interpretation of the study results more comprehensive and accurate.
3. The study covered a variety of treatments, including biologics (Dupilumab, Tralokinumab), traditional systemic medications, topical medications (Pimecrolimus), and emerging therapies (probiotics, melatonin). By including different therapies, the study provides clinicians with more treatment options, especially in cases where the response to different treatment options may vary in different patient populations and can provide a valuable reference for physicians.

Although this study integrated multiple studies through a web-based Meta-analysis approach, there was some heterogeneity among the included studies. For example, the design, baseline patient characteristics, disease severity, and specific use of treatment regimens may differ across studies, and these differences may affect the accuracy and generalizability of the results. Some studies may have focused only on specific subgroups (patients with severe disease or adult patients), and the response to treatment in these subgroups may have differed from that in the pediatric population in this study, which in turn may have affected the broad applicability of the treatment effects. In addition, most of the included studies in this study focused only on short-term efficacy and lacked long-term follow-up data, resulting in an inability to adequately assess the long-term effects and potential side effects of the treatments. Atopic dermatitis, as a chronic disease, the long-term effects of treatment are crucial, and the lack of long-term data makes a comprehensive evaluation of treatment options limited. Therefore, future studies should focus on these heterogeneity issues and enhance the assessment of long-term efficacy and safety.

An important methodological consideration is that several dupilumab and JAK inhibitor trials mandated concomitant topical corticosteroids, whereas topical agents such as pimecrolimus were typically evaluated as monotherapy against vehicle controls. This heterogeneity in background therapy may have influenced treatment effect estimates and indirect comparisons within the network. Specifically, the additive effect of TCS in biologic or JAK inhibitor trials may confound comparisons with topical monotherapies, potentially affecting ranking probabilities for outcomes such as EASI improvement. Therefore, the observed superiority of pimecrolimus in EASI ranking should be interpreted cautiously.

## Conclusion

This study compared the efficacy of various treatment regimens in pediatric atopic dermatitis by network Meta-analysis and found significant differences in the relief of skin symptoms among different treatments. The results showed that Dupilumab was significantly superior to other treatments, such as melatonin, probiotics, SCG, Synbiotic, and VD, in terms of improvement in SCORAD and EASI scores. Tralokinumab, PEC, and other therapeutic regimens also demonstrated better efficacy. Nonetheless, there are still some limitations of the studies, including heterogeneity of included studies and lack of long-term follow-up data, which failed to comprehensively assess the long-term effects of the treatments and potential side effects. Future studies should focus on addressing these issues, especially by designing more consistent clinical trials to further evaluate the long-term efficacy and safety of the treatments, to provide more precise clinical guidance. Therefore, although this study provides abundant short-term efficacy data, more long-term follow-up and safety data are needed for a comprehensive evaluation of atopic dermatitis treatments.

## Data Availability

The original contributions presented in the study are included in the article/**Supplementary Material**. Further inquiries can be directed to the corresponding authors.

## References

[1] Chiesa Fuxench ZC Mitra N Del Pozo D Hoffstad O Shin DB Margolis DJ . Risk of atopic dermatitis and the atopic march paradigm in children of mothers with atopic illnesses: a birth cohort study from the United Kingdom. J Am Acad Dermatol. (2024) 90:561–8. doi: 10.1016/j.jaad.2023.11.013. PMID: 37984723 PMC10922528

[2] Halling AS Thyssen JP . Biological therapy for young children with atopic dermatitis. Lancet. (2022) 400:867–9. doi: 10.1016/s0140-6736(22)01742-1. PMID: 36116468

[3] Fuxench ZCC Wan J Wang S Syed MN Shin DB Abuabara K . Atopic dermatitis and risk for headache disorders and migraines: a population-based cohort study in children and adults from the UK. Br J Dermatol. (2023) 190:120–3. doi: 10.1093/bjd/ljad325. PMID: 37671663

[4] Hu C Jansen PW . Do adolescents with atopic dermatitis have lower cognitive function and school performance than their peers without atopic dermatitis? Br J Dermatol. (2023) 188:313–9. doi: 10.1093/bjd/ljac105. PMID: 36637155

[5] Tsai SY Gaffin JM Hawryluk EB Ruran HB Bartnikas LM Oyoshi MK . Evaluation of dupilumab on the disease burden in children and adolescents with atopic dermatitis: a population-based cohort study. Allergy. (2024) 79:2748–58. doi: 10.1111/all.16265. PMID: 39166365 PMC11608558

[6] Vestergaard C . The complex relationship between maternal smoking and atopic dermatitis in children. J Eur Acad Dermatol Venereol. (2024) 38:1840–1. doi: 10.1111/jdv.20260. PMID: 39319940

[7] Wan J Shin DB Syed MN Abuabara K Lemeshow AR Gelfand JM . Atopic dermatitis and risk of major neuropsychiatric disorders in children: a population-based cohort study. J Eur Acad Dermatol Venereol. (2023) 37:114–22. doi: 10.1111/jdv.18564. PMID: 36018560 PMC9929490

[8] Cheng BT Paller AS Griffith JW Silverberg JI Fishbein AB . Burden and characteristics of skin pain among children with atopic dermatitis. J Allergy Clin Immunol Pract. (2022) 10:1104–1106.e1. doi: 10.1016/j.jaip.2021.12.012. PMID: 34954412 PMC9275595

[9] Wollenberg A Werfel T Ring J Ott H Gieler U Weidinger S . Atopic dermatitis in children and adults—diagnosis and treatment. Dtsch Arztebl Int. (2023) 120:224–34. doi: 10.1007/978-3-540-78814-0_23. PMID: 36747484 PMC10277810

[10] Ahn J Shin S Lee GC Han BE Lee E Ha EK . Unraveling the link between atopic dermatitis and autoimmune diseases in children: insights from a large-scale cohort study with 15-year follow-up and shared gene ontology analysis. Allergol Int. (2024) 73:243–54. doi: 10.1016/j.alit.2023.12.005. PMID: 38238236

[11] Mohammad S Karim MR Iqbal S Lee JH Mathiyalagan R Kim YJ . Atopic dermatitis: pathophysiology, microbiota, and metabolome - a comprehensive review. Microbiol Res. (2024) 281:127595. doi: 10.1016/j.micres.2023.127595. PMID: 38218095

[12] Weins AB Kerzel S Schnopp C . Severe atopic dermatitis in early infancy: characteristics, challenges and new perspectives in clinical practice. J Dtsch Dermatol Ges. (2024) 22:350–5. doi: 10.1111/ddg.15344. PMID: 38450908

[13] Maleki-Yazdi KA Heen AF Zhao IX Guyatt GH Suzumura EA Makhdami N . Values and preferences of patients and caregivers regarding treatment of atopic dermatitis (eczema): a systematic review. JAMA Dermatol. (2023) 159:320–30. doi: 10.1016/j.anai.2022.08.717. PMID: 36696136

[14] Paller AS de Bruin-Weller M Marcoux D Baselga E Oliveira de Carvalho V Ardusso LRF . Real-world treatment outcomes of systemic treatments for moderate-to-severe atopic dermatitis in children aged less than 12 years: 2-year results from PEDIatric STudy in Atopic Dermatitis. J Am Acad Dermatol. (2025) 92:242–51. doi: 10.1016/j.jaad.2024.09.046. PMID: 39389429

[15] Stölzl D Sander N Siegels D Harder I Kind B Fonfara M . Clinical and molecular response to dupilumab treatment in pediatric atopic dermatitis: results of the German TREATkids registry. Allergy. (2024) 79:2849–52. 10.1111/all.16147 38712730

[16] Flohr C . How we treat atopic dermatitis now and how that will change over the next 5 years. Br J Dermatol. (2023) 188:718–25. doi: 10.1093/bjd/ljac116. PMID: 36715500

[17] Rothenberg-Lausell C Bar J Dahabreh D Renert-Yuval Y Del Duca E Guttman-Yassky E . Biologic and small-molecule therapy for treating moderate to severe atopic dermatitis: mechanistic considerations. J Allergy Clin Immunol. (2024) 154:20–30. doi: 10.1016/j.jaci.2024.04.009. PMID: 38670231

[18] Ryan Wolf J Chen A Wieser J Johnson B Baughman L Lee G . Improved patient- and caregiver-reported outcomes distinguish tacrolimus 0.03% from crisaborole in children with atopic dermatitis. J Eur Acad Dermatol Venereol. (2024) 38:1364–72. doi: 10.1111/jdv.19807. PMID: 38357778 PMC11209823

[19] Page MJ McKenzie JE Bossuyt PM Boutron I Hoffmann TC Mulrow CD . The PRISMA 2020 statement: an updated guideline for reporting systematic reviews. Bmj. (2021) 372:n71. doi: 10.31222/osf.io/v7gm2. PMID: 33782057 PMC8005924

[20] Lu T Lu C Li H Xing X Deng X Li X . The reporting quality and risk of bias of randomized controlled trials of acupuncture for migraine: methodological study based on STRICTA and RoB 2.0. Compl Ther Med. (2020) 52:102433. doi: 10.1016/j.ctim.2020.102433. PMID: 32951707

[21] Nikolakopoulou A Higgins JPT Papakonstantinou T Chaimani A Del Giovane C Egger M . CINeMA: an approach for assessing confidence in the results of a network meta-analysis. PloS Med. (2020) 17:e1003082. doi: 10.1371/journal.pmed.1003082. PMID: 32243458 PMC7122720

[22] Papakonstantinou T Nikolakopoulou A Higgins JPT Egger M Salanti G . CINeMA: software for semiautomated assessment of the confidence in the results of network meta-analysis. Campb Syst Rev. (2020) 16:e1080. doi: 10.1002/cl2.1080. PMID: 37131978 PMC8356302

[23] Aziz MK Molony D Monlezun D Holder T Brunckhorst O Higgason N . Prostate cancer therapy cardiotoxicity map (PROXMAP) for advanced disease states: a systematic review and network meta-analysis with Bayesian modeling of treatment histories. Eur Urol. (2025) 87:15–26. doi: 10.1016/j.eururo.2024.08.031. PMID: 39299896

[24] Abbasi S Kamalinejad M Babaie D Shams S Sadr Z Gheysari M . A new topical treatment of atopic dermatitis in pediatric patients based on Ficus carica L. (Fig): a randomized, placebo-controlled clinical trial. Compl Ther Med. (2017) 35:85–91. doi: 10.1016/j.ctim.2017.10.003. PMID: 29154073

[25] Ahn SH Yoon W Lee SY Shin HS Lim MY Nam YD . Effects of Lactobacillus pentosus in children with allergen-sensitized atopic dermatitis. J Kor Med Sci. (2020) 35:e128. doi: 10.3346/jkms.2020.35.e128. PMID: 32383366 PMC7211510

[26] Ardakani AT Farrehi M Sharif MR Ostadmohammadi V Mirhosseini N Kheirkhah D . The effects of melatonin administration on disease severity and sleep quality in children with atopic dermatitis: a randomized, double-blinded, placebo-controlled trial. Pediatr Allergy Immunol. (2018) 29:834–40. doi: 10.1111/pai.12978. PMID: 30160043

[27] Berth-Jones J Pollock I Hearn RM Lewis-Jones S Goodfield M Griffiths CE . A randomised, controlled trial of a 4% cutaneous emulsion of sodium cromoglicate in treatment of atopic dermatitis in children. J Dermatol Treat. (2015) 26:291–6. doi: 10.3109/09546634.2014.946380. PMID: 25034003

[28] Borzutzky A Iturriaga C Pérez-Mateluna G Cristi F Cifuentes L Silva-Valenzuela S . Effect of weekly vitamin D supplementation on the severity of atopic dermatitis and type 2 immunity biomarkers in children: a randomized controlled trial. J Eur Acad Dermatol Venereol. (2024) 38:1760–8. doi: 10.1111/jdv.19959. PMID: 38483248

[29] Chan S Cornelius V Cro S Harper JI Lack G . Treatment effect of omalizumab on severe pediatric atopic dermatitis: the ADAPT randomized clinical trial. JAMA Pediatr. (2020) 174:29–37. doi: 10.1001/jamapediatrics.2019.4476. PMID: 31764962 PMC6902112

[30] Chang YS Lin MH Lee JH Lee PL Dai YS Chu KH . Melatonin supplementation for children with atopic dermatitis and sleep disturbance: a randomized clinical trial. JAMA Pediatr. (2016) 170:35–42. doi: 10.2165/00128071-200203030-00004. PMID: 26569624

[31] Edwards AM Bibawy D Matthews S Tongue N Arshad SH Lødrup Carlsen K . Long-term use of a 4% sodium cromoglicate cutaneous emulsion in the treatment of moderate to severe atopic dermatitis in children. J Dermatol Treat. (2015) 26:541–7. doi: 10.3109/09546634.2015.1034077. PMID: 25909369

[32] Eichenfield LF Lucky AW Boguniewicz M Langley RG Cherill R Marshall K . Safety and efficacy of pimecrolimus (ASM 981) cream 1% in the treatment of mild and moderate atopic dermatitis in children and adolescents. J Am Acad Dermatol. (2002) 46:495–504. doi: 10.1067/mjd.2002.122187. PMID: 11907497

[33] Farid R Ahanchian H Jabbari F Moghiman T . Effect of a new synbiotic mixture on atopic dermatitis in children: a randomized-controlled trial. Iran J Pediatr. (2011) 21:225–30. PMC3446166 23056792

[34] Feíto-Rodríguez M Ramírez-Boscà A Vidal-Asensi S Fernández-Nieto D Ros-Cervera G Alonso-Usero V . Randomized double-blind placebo-controlled clinical trial to evaluate the effect of a mixture of probiotic strains on symptom severity and use of corticosteroids in children and adolescents with atopic dermatitis. Clin Exp Dermatol. (2023) 48:495–503. 36637147 10.1093/ced/llad007

[35] Fölster-Holst R Müller F Schnopp N Abeck D Kreiselmaier I Lenz T . Prospective, randomized controlled trial on Lactobacillus rhamnosus in infants with moderate to severe atopic dermatitis. Br J Dermatol. (2006) 155:1256–61. doi: 10.1111/j.1365-2133.2006.07558.x. PMID: 17107398

[36] Gerasimov SV Vasjuta VV Myhovych OO Bondarchuk LI . Probiotic supplement reduces atopic dermatitis in preschool children: a randomized, double-blind, placebo-controlled, clinical trial. Am J Clin Dermatol. (2010) 11:351–61. doi: 10.2165/11531420-000000000-00000. PMID: 20642296

[37] Grüber C Wendt M Sulser C Lau S Kulig M Wahn U . Randomized, placebo-controlled trial of Lactobacillus rhamnosus GG as treatment of atopic dermatitis in infancy. Allergy. (2007) 62:1270–6. 10.1111/j.1398-9995.2007.01543.x 17919141

[38] Han Y Kim B Ban J Lee J Kim BJ Choi BS . A randomized trial of Lactobacillus plantarum CJLP133 for the treatment of atopic dermatitis. Pediatr Allergy Immunol. (2012) 23:667–73. doi: 10.1016/j.det.2018.07.003. PMID: 23050557

[39] Jeong K Kim M Jeon SA Kim YH Lee S . A randomized trial of Lactobacillus rhamnosus IDCC 3201 tyndallizate (RHT3201) for treating atopic dermatitis. Pediatr Allergy Immunol. (2020) 31:783–92. doi: 10.1111/pai.13269. PMID: 32363613

[40] Lara-Corrales I Huang CM Parkin PC Rubio-Gomez GA Posso-De Los Rios CJ Maguire J . Vitamin D level and supplementation in pediatric atopic dermatitis: a randomized controlled trial. J Cutan Med Surg. (2019) 23:44–9. doi: 10.1177/1203475418805744. PMID: 30336685

[41] Meysami M Hashempur MH Kamalinejad M Emtiazy M . Efficacy of short term topical Malva sylvestris L. cream in pediatric patients with atopic dermatitis: a randomized double-blind placebo-controlled clinical trial. Endocr Metab Immune Disord Drug Targets. (2021) 21:1673–8. doi: 10.2174/1871530320666201023125411. PMID: 33100212

[42] Mirrahimi B Moazemi M Eslami N Jamshidi E Mir M Mohebbi R . Evaluating the effect of eicosapentaenoic acid in children with atopic dermatitis: a randomized triple-blind clinical trial. J Pediatr Pharmacol Ther. (2023) 28:29–35. doi: 10.5863/1551-6776-28.1.29. PMID: 36777980 PMC9901318

[43] Navarro-López V Ramírez-Boscà A Ramón-Vidal D Ruzafa-Costas B Genovés-Martínez S Chenoll-Cuadros E . Effect of oral administration of a mixture of probiotic strains on SCORAD index and use of topical steroids in young patients with moderate atopic dermatitis: a randomized clinical trial. JAMA Dermatol. (2018) 154:37–43. doi: 10.1001/jamadermatol.2017.3647. PMID: 29117309 PMC5833582

[44] Niseteo T Hojsak I Bulic SO Pustisek N . Effect of omega-3 polyunsaturated fatty acid supplementation on clinical outcome of atopic dermatitis in children. Nutrients. (2024) 16:1–12. doi: 10.20944/preprints202407.1619.v1. PMID: 39275147 PMC11397185

[45] Paller AS Pinter A Lee LW Aschoff R Zdybski J Schnopp C . Efficacy and safety of dupilumab treatment with concomitant topical corticosteroids in children aged 6 months to 5 years with severe atopic dermatitis. Adv Ther. (2024) 41:1046–61. doi: 10.1016/j.reval.2023.103411. PMID: 38194047 PMC10879230

[46] Saeki H Baba N Oshiden K Abe Y Tsubouchi H . Phase 2, randomized, double-blind, placebo-controlled, 4-week study to evaluate the safety and efficacy of OPA- 15406 (difamilast), a new topical selective phosphodiesterase type-4 inhibitor, in Japanese pediatric patients aged 2–14 years with atopic dermatitis. J Dermatol. (2020) 47:17–24. doi: 10.1111/1346-8138.15137. PMID: 31713267 PMC6972691

[47] Shafiei A Moin M Pourpak Z Gharagozlou M Aghamohamadi A Sajedi V . Synbiotics could not reduce the scoring of childhood atopic dermatitis (SCORAD): a randomized double blind placebo-controlled trial. Iran J Allergy Asthma Immunol. (2011) 10:21–8. 21358011

[48] Simpson EL Paller AS Siegfried EC Boguniewicz M Sher L Gooderham MJ . Efficacy and safety of dupilumab in adolescents with uncontrolled moderate to severe atopic dermatitis a phase 3 randomized clinical trial. JAMA Dermatol. (2020) 156:44–56. doi: 10.1001/jamadermatol.2019.3336. PMID: 31693077 PMC6865265

[49] Torrelo A Rewerska B Galimberti M Paller A Yang CY Prakash A . Efficacy and safety of baricitinib in combination with topical corticosteroids in paediatric patients with moderate-to-severe atopic dermatitis with an inadequate response to topical corticosteroids: results from a phase III, randomized, double-blind, placebo-controlled study (BREEZE-AD PEDS). Br J Dermatol. (2023) 189:23–32. doi: 10.1093/bjd/ljad096. PMID: 36999560

[50] Wu YJ Wu WF Hung CW Ku MS Liao PF Sun HL . Evaluation of efficacy and safety of Lactobacillus rhamnosus in children aged 4–48 months with atopic dermatitis: an 8-week, double-blind, randomized, placebo-controlled study. J Microbiol Immunol Infect. (2017) 50:684–92. doi: 10.1016/j.jmii.2015.10.003. PMID: 26733351

[51] Yang HJ Min TK Lee HW Pyun BY . Efficacy of probiotic therapy on atopic dermatitis in children: a randomized, double-blind, placebo-controlled trial. Allergy Asthma Immunol Res. (2014) 6:208–15. doi: 10.4168/aair.2014.6.3.208. PMID: 24843795 PMC4021238

[52] Igarashi A Katsunuma T Matsumura T Komazaki H . Efficacy and safety of nemolizumab in paediatric patients aged 6–12 years with atopic dermatitis with moderate-to-severe pruritus: results from a phase III, randomized, double-blind, placebo-controlled, multicentre study. Br J Dermatol. (2024) 190:20–8. doi: 10.1093/bjd/ljad268. PMID: 37522351

[53] Paller AS Flohr C Cork M Bewley A Blauvelt A Hong HCH . Efficacy and safety of tralokinumab in adolescents with moderate to severe atopic dermatitis: the phase 3 ECZTRA 6 randomized clinical trial. JAMA Dermatol. (2023) 159:596–605. doi: 10.1001/jamadermatol.2023.0627. PMID: 37074705 PMC10116386

[54] Van Der Aa LB Heymans HS Van Aalderen WM Sillevis Smitt JH Knol J Ben Amor K . Effect of a new synbiotic mixture on atopic dermatitis in infants: a randomized-controlled trial. Clin Exp Allergy. (2010) 40:795–804. doi: 10.1111/j.1365-2222.2010.03465.x. PMID: 20184604

[55] Eichenfield LF Flohr C Sidbury R Siegfried E Szalai Z Galus R . Efficacy and safety of abrocitinib in combination with topical therapy in adolescents with moderate-to-severe atopic dermatitis: the JADE TEEN randomized clinical trial. JAMA Dermatol. (2021) 157:1165–73. doi: 10.1001/jamadermatol.2021.2830. PMID: 34406366 PMC8374743

[56] Blauvelt A Ladizinski B Prajapati VH Laquer V Fischer A Eisman S . Efficacy and safety of switching from dupilumab to upadacitinib versus continuous upadacitinib in moderate-to-severe atopic dermatitis: results from an open-label extension of the phase 3, randomized, controlled trial (Heads Up). J Am Acad Dermatol. (2023) 89:478–85. doi: 10.1016/j.jaad.2023.05.033. PMID: 37230366

[57] Hasan I Parsons L Duran S Zinn Z . Dupilumab therapy for atopic dermatitis is associated with increased risk of cutaneous T cell lymphoma: a retrospective cohort study. J Am Acad Dermatol. (2024) 91:255–8. doi: 10.1016/j.jaad.2024.03.039. PMID: 38588818

[58] Wollenberg A Beck LA Blauvelt A Simpson EL Chen Z Chen Q . Laboratory safety of dupilumab in moderate-to-severe atopic dermatitis: results from three phase III trials (LIBERTY AD SOLO 1, LIBERTY AD SOLO 2, LIBERTY AD CHRONOS). Br J Dermatol. (2020) 182:1120–35. doi: 10.1111/bjd.18434. PMID: 31407311 PMC7317598

[59] Beck LA Bieber T Weidinger S Tauber M Saeki H Irvine AD . Tralokinumab treatment improves the skin microbiota by increasing the microbial diversity in adults with moderate-to-severe atopic dermatitis: analysis of microbial diversity in ECZTRA 1, a randomized controlled trial. J Am Acad Dermatol. (2023) 88:816–23. doi: 10.1016/j.jaad.2022.11.047. PMID: 36473633

[60] Chu CY Yao TC Shih IH Yang CY Chin CL Ibrahim S . Pimecrolimus for the treatment of atopic dermatitis in infants: an Asian perspective. Dermatol Ther (Heidelb). (2023) 13:717–27. doi: 10.1007/s13555-022-00886-9. PMID: 36735214 PMC9984644

[61] Sun S Chang G Zhang L . The prevention effect of probiotics against eczema in children: an update systematic review and meta-analysis. J Dermatol Treat. (2022) 33:1844–54. doi: 10.1080/09546634.2021.1925077. PMID: 34006167

[62] Ardern-Jones MR Brown SJ Flohr C Hossain P Irvine AD Johnston GA . An expert consensus on managing dupilumab-related ocular surface disorders in people with atopic dermatitis 2024. Br J Dermatol. (2024) 191:865–85. doi: 10.1093/bjd/ljae344. PMID: 39236226

[63] Simpson EL Guttman-Yassky E Eichenfield LF Boguniewicz M Bieber T Schneider S . Tralokinumab therapy for moderate-to-severe atopic dermatitis: clinical outcomes with targeted IL-13 inhibition. Allergy. (2023) 78:2875–91. doi: 10.1111/all.15811. PMID: 37455359

[64] Luger T Chu CY Elgendy A Ibrahim S Murashkin N Ranjan S . Pimecrolimus 1% cream for mild-to-moderate atopic dermatitis: a systematic review and meta-analysis with a focus on children and sensitive skin areas. Eur J Dermatol. (2023) 33:474–86. doi: 10.1684/ejd.2023.4556. PMID: 38297923

[65] Zhao S Hwang A Miller C Lio P . Safety of topical medications in the management of paediatric atopic dermatitis: an updated systematic review. Br J Clin Pharmacol. (2023) 89:2039–65. doi: 10.1111/bcp.15751. PMID: 37075252

