# Supplementary material for: Comparative efficacy of pediatric atopic dermatitis treatments: a network meta-analysis highlighting dupilumab and pimecrolimus for SCORAD and EASI improvement
Source: Front Immunol. 2026 May 1;17:1676852. doi: 10.3389/fimmu.2026.1676852 (PMC13176288; doi:10.3389/fimmu.2026.1676852)
Supplement: Supplementary file 1 [file DataSheet1.docx]

Table S1 retrieval strategy

((((("Child"[Mesh]) OR ((Child[MeSH Terms]) OR (Children[Title/Abstract]))) OR ("Pediatrics"[Mesh])) OR (Pediatrics[MeSH Terms])) AND (("Dermatitis, Atopic"[Mesh]) OR ((((((((((Dermatitis, Atopic[MeSH Terms]) OR (Atopic Dermatitis[Title/Abstract])) OR (Eczema, Atopic[Title/Abstract])) OR (Atopic Eczema[Title/Abstract])) OR (Neurodermatitis, Atopic[Title/Abstract])) OR (Atopic Neurodermatitis[Title/Abstract])) OR (Neurodermatitis, Disseminated[Title/Abstract])) OR (Disseminated Neurodermatitis[Title/Abstract])) OR (Eczema, Infantile[Title/Abstract])) OR (Infantile Eczema[Title/Abstract])))) AND (randomized controlled trial[Publication Type] OR randomized[Title/Abstract] OR placebo[Title/Abstract])

Table S2 certainty of the evidence

| Comparison | Number of studies | Within-study bias | Reporting bias | Indirectness | Imprecision | Heterogeneity | Incoherence | Confidence rating | Reason(s) for downgrading |
| --- | --- | --- | --- | --- | --- | --- | --- | --- | --- |
| Control:Dupilumab | 3 | No concerns | Some concerns | No concerns | No concerns | No concerns | Major concerns | Low | ["Reporting bias","Incoherence"] |
| Control:EPA | 2 | No concerns | Some concerns | No concerns | No concerns | Major concerns | Major concerns | Very low | ["Reporting bias","Heterogeneity","Incoherence"] |
| Control:Melatonin | 2 | No concerns | Some concerns | No concerns | No concerns | Major concerns | Major concerns | Very low | ["Reporting bias","Heterogeneity","Incoherence"] |
| Control:Omalizumab | 1 | No concerns | Some concerns | No concerns | Major concerns | No concerns | Major concerns | Low | ["Reporting bias","Imprecision","Incoherence"] |
| Control:PEC | 2 | No concerns | Some concerns | No concerns | No concerns | No concerns | Major concerns | Moderate | ["Reporting bias","Incoherence"] |
| Control:Probiotics | 9 | No concerns | Some concerns | No concerns | No concerns | Major concerns | Major concerns | Very low | ["Reporting bias","Heterogeneity","Incoherence"] |
| Control:SCG | 2 | No concerns | Some concerns | No concerns | Major concerns | No concerns | Major concerns | Very low | ["Reporting bias","Imprecision","Incoherence"] |
| Control:Synbiotic | 3 | Some concerns | Some concerns | No concerns | Some concerns | Some concerns | Major concerns | Low | ["Within-study bias","Reporting bias","Imprecision","Heterogeneity","Incoherence"] |
| Control:Tralokinumab | 2 | No concerns | Some concerns | No concerns | No concerns | No concerns | Major concerns | Low | ["Reporting bias","Incoherence"] |
| Control:VD | 2 | Some concerns | Some concerns | No concerns | Major concerns | No concerns | Major concerns | Low | ["Within-study bias","Reporting bias","Imprecision","Incoherence"] |
| EASI | | | | | | | | | |
| Comparison | Number of studies | Within-study bias | Reporting bias | Indirectness | Imprecision | Heterogeneity | Incoherence | Confidence rating | Reason(s) for downgrading |
| Abrocitinib:Control | 2 | No concerns | Some concerns | No concerns | No concerns | No concerns | Major concerns | Moderate | ["Reporting bias","Incoherence"] |
| Baricitinib:Control | 3 | No concerns | Some concerns | No concerns | No concerns | No concerns | Major concerns | Moderate | ["Reporting bias","Incoherence"] |
| Control:Difamilast | 1 | No concerns | Some concerns | No concerns | No concerns | No concerns | Major concerns | Moderate | ["Reporting bias","Incoherence"] |
| Control:Dupilumab | 2 | No concerns | Some concerns | No concerns | No concerns | No concerns | Major concerns | Moderate | ["Reporting bias","Incoherence"] |
| Control:Nemolizumab | 1 | No concerns | Some concerns | No concerns | No concerns | No concerns | Major concerns | Moderate | ["Reporting bias","Incoherence"] |
| Control:Omalizumab | 1 | No concerns | Some concerns | No concerns | No concerns | Major concerns | Major concerns | Low | ["Reporting bias","Heterogeneity","Incoherence"] |
| Control:Pimecrolimus | 1 | No concerns | Some concerns | No concerns | No concerns | No concerns | Major concerns | Moderate | ["Reporting bias","Incoherence"] |
| Control:Probiotics | 1 | No concerns | Some concerns | No concerns | Major concerns | No concerns | Major concerns | Low | ["Reporting bias","Imprecision","Incoherence"] |
| Control:Tralokinumab | 2 | No concerns | Some concerns | No concerns | No concerns | No concerns | Major concerns | Moderate | ["Reporting bias","Incoherence"] |

PEC: Plant extract cream; SCG: Sodium Cromoglicate; VD: vitamin D; EPA: Eicosapentaenoic Acid; SCORAD: scoring atopic dermatitis; EASI: Eczema Area and Severity Index.

| Outcomes | Consistency modeling | Inconsistency modeling |
| --- | --- | --- |
| SCORAD | 110.21 | 110.35 |
| EASI | 51.84 | 51.76 |

Table s3 Results of consistency modeling

SCORAD: scoring atopic dermatitis; EASI: Eczema Area and Severity Index.


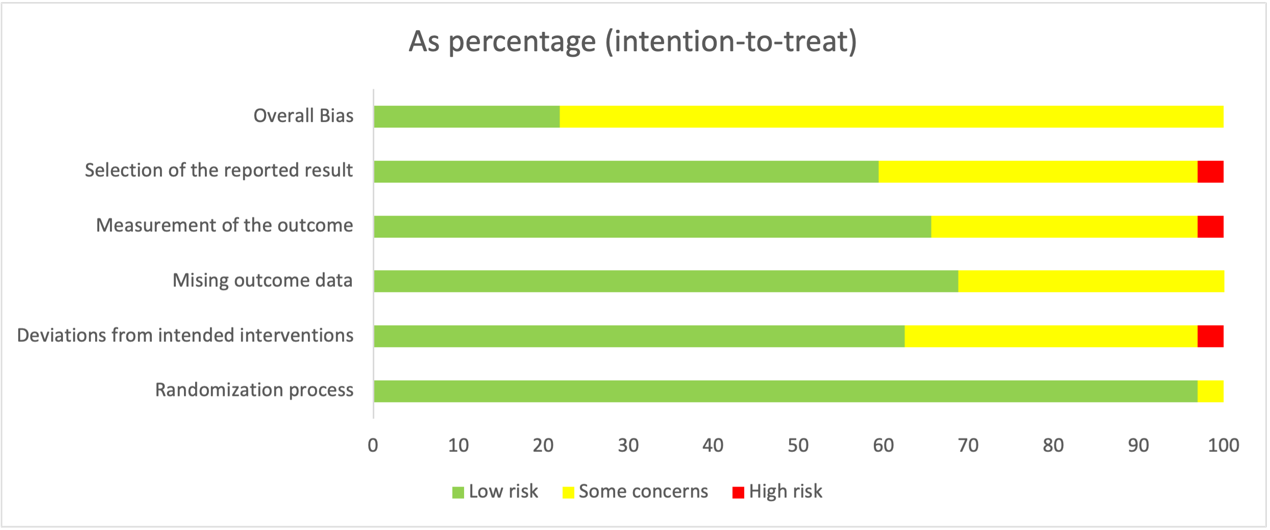


Figure S1 risk of bias graph


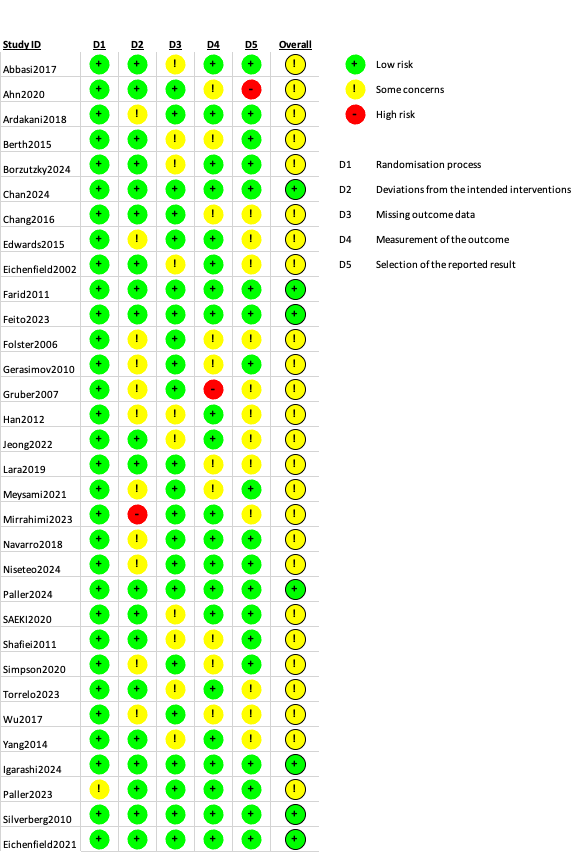


Figure S2 risk of bias summary


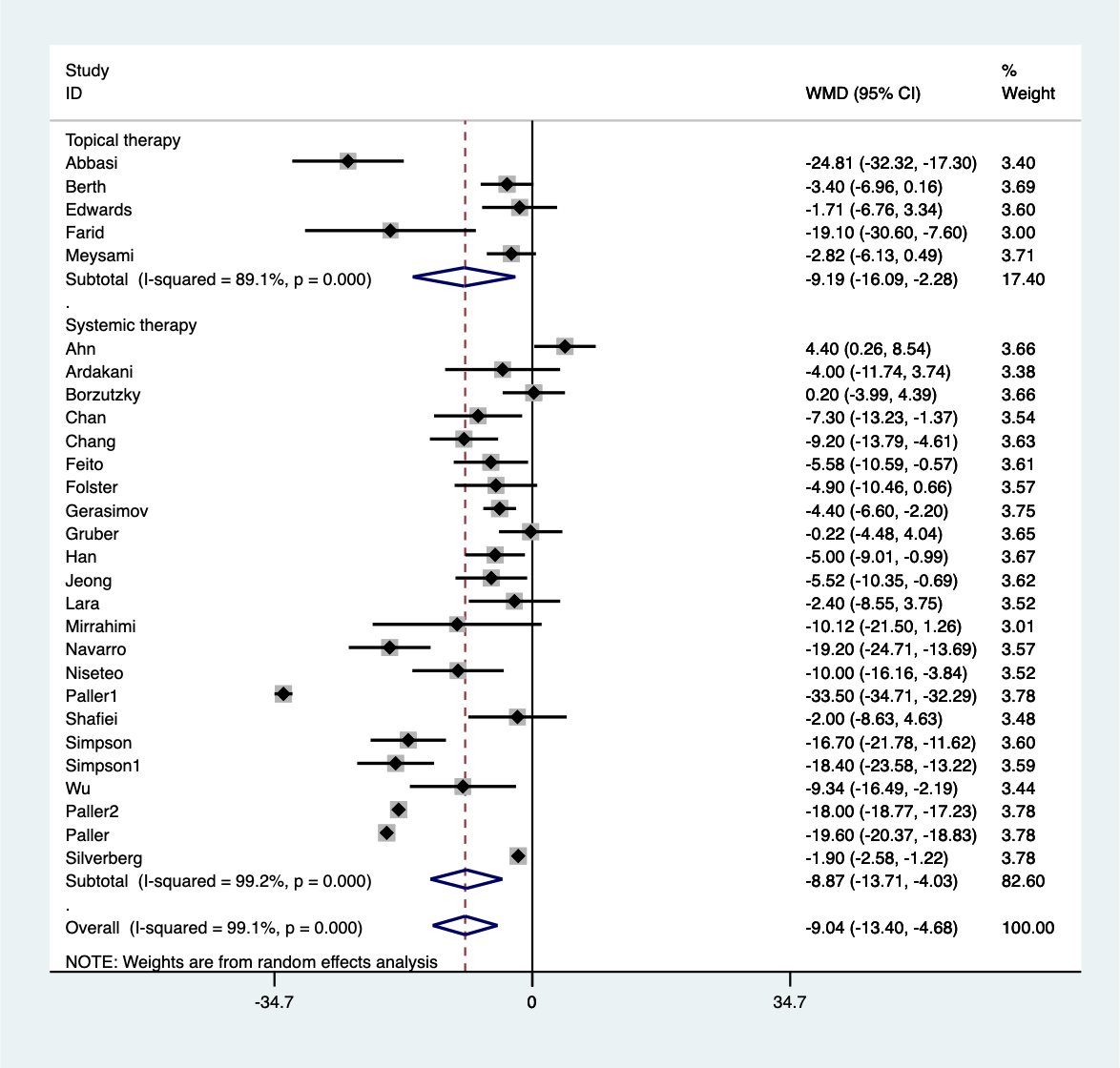


Figure S3 Subgroup analysis of SCORAD treatment regimens


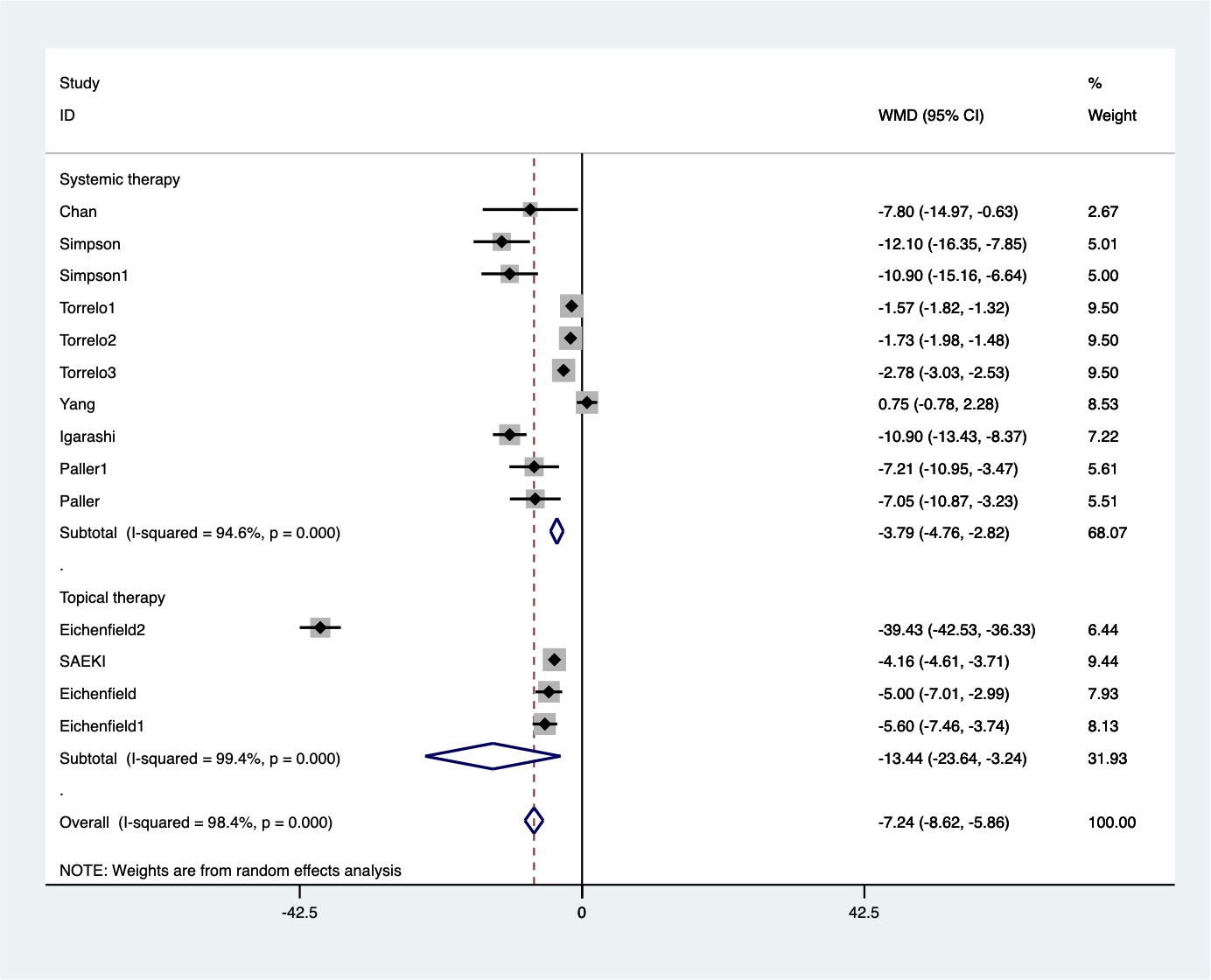


Figure S4 Subgroup analysis of EASI treatment regimens


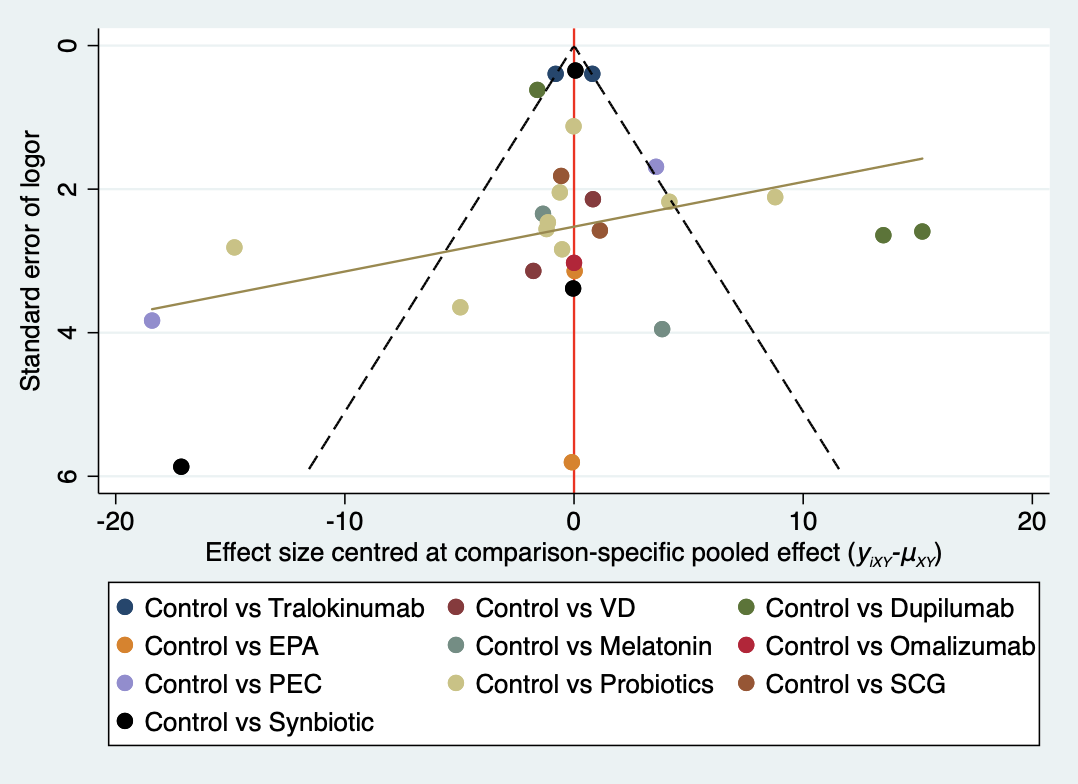


Figure S5 SCORAD funnel plot


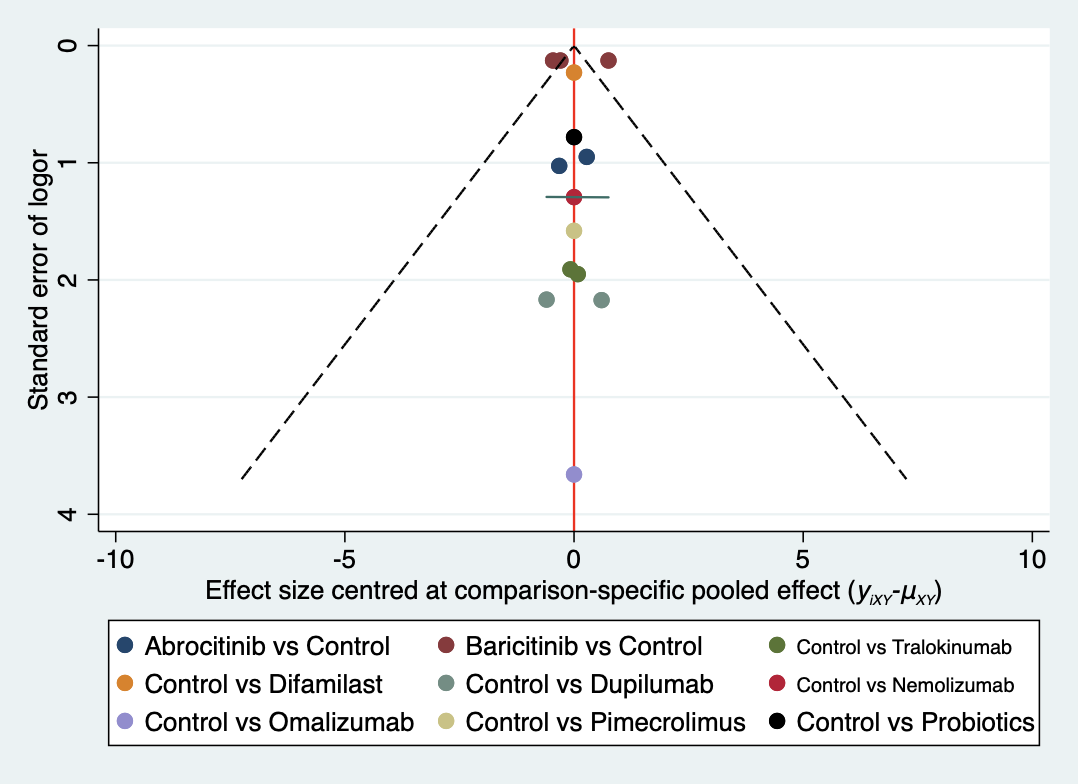


Figure S6 EASI funnel plot
